# History of postgraduate psychiatry education and training in Nigeria

**DOI:** 10.4102/sajpsychiatry.v27.i0.1651

**Published:** 2021-05-28

**Authors:** Muideen O. Bakare

**Affiliations:** 1 Child and Adolescent Unit, Clinical Services, Federal Neuropsychiatric Hospital, Enugu, Nigeria; 2 Department of Research and Advocacy, Childhood Neuropsychiatric Disorders Initiatives (CNDI), Enugu, Nigeria

## Introduction

The history of western medical education and postgraduate psychiatry training has been sparsely documented in Nigeria. This article explores this topic in a succinct and systematic manner for the purpose of future documentation and referencing. This history is documented and updated up to January 2021. Nigeria, also known as the Federal Republic of Nigeria, is located in the West African sub-region bordered by the Republic of Benin in the West, Chad and Cameroon in the East and the Niger Republic in the North. The Nigerian coast in the South lies on the Gulf of Guinea in the Atlantic Ocean. Nigeria has more than 250 ethnic groups, but the three major traditional groups are the Hausa-Fulani, the Yoruba and the Igbo. Geopolitically, the country consists of six regions or zones, 36 states and a Federal Capital Territory located in Abuja. It consists of about 20% of the sub-Saharan African population.^1^

Early western medical training in Nigeria prior to 1932 was mostly acquired in the United Kingdom (UK), few other European countries and the United States of America. Some Nigerians also obtained scholarship to study medicine in Eastern Europe and Russia up to 1960.Indigenous Western medical training commenced in Nigeria in 1932 when the Yaba Higher College provided a 7-year basic medical education course for candidates with Senior Cambridge School Certificate and produced assistant medical officers whose certificates were only recognised in Nigeria. The assistant medical officers were like physician assistants as observed today in the United States of America.^2^ This course spanned the period 1932–1952, after which it was phased out for a more preferable course at the Faculty of Medicine, University of Ibadan, a programme that has a special relationship with the University of London where students who completed the pre-clinical training often proceed for the clinical aspect of their training.^2^

Most of these foreign trained doctors remained in the UK to acquire postgraduate training in different specialties of medicine. Those who specialised in psychiatry then came back with Diploma in Psychological Medicine. These groups of doctors became the ‘founding fathers’ as they are fondly referred to forming the foundation of indigenous postgraduate psychiatry training in Nigeria and they went ahead to train other psychiatrists in Nigeria.^2^ Notable amongst them are late Prof. Thomas Adeoye Lambo, the first Nigerian psychiatrist who later rose in his carrier to become a deputy director at the World Health Organization (WHO).^3^ Others include the likes of late Prof. Tolani Asuni, who is the second indigenous psychiatrist in Nigeria.^4^ Closely followed these two are Prof. Ayo Binitie and Prof. Betta Johnson, amongst others.^5^

## Inception of postgraduate medical training in Nigeria

**FIGURE 1:**
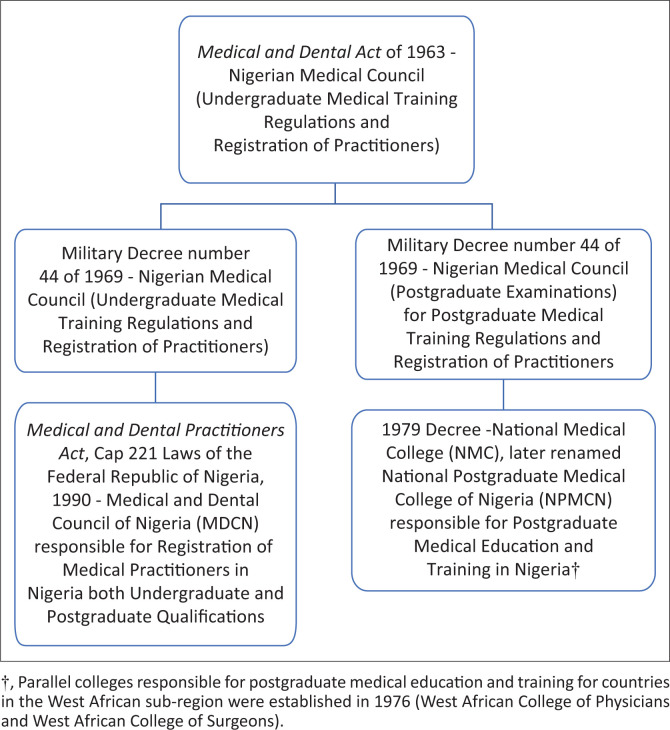
Evolution of postgraduate medical training in Nigeria of which psychiatry is a component.

Military Decree number 44 of 1969 gave a take-off wing to indigenous postgraduate medical training in Nigeria after due approval by the then Nigerian Medical Council (Figure 1). This decree was an amendment of the *Medical and Dental Practitioners Act* of 1963.^2^ The Nigerian Medical Council later became Medical and Dental Council of Nigeria (MDCN) in 1990 through the promulgation of the *Medical and Dental Practitioners Act*, Cap 221 Laws of the Federal Republic of Nigeria, 1990.^6^ *Medical and Dental Act* of 1963 was the law regulating medical graduate registration and practice. An amendment to it led to Decree number 44 of 1969 to enable the Nigerian Medical Council to be also responsible for postgraduate medical training and registration/practice of postgraduate medicine as opposed to only undergraduate medical training.

Postgraduate medical training including psychiatry was superintended by the then Nigerian Medical Council (Postgraduate Examinations), the section of Nigerian Medical Council that assumed the responsibility of postgraduate medical education with the support of external examiners from the UK and neighbouring West African countries from 1970 up to 1979, when postgraduate training was separated under an independent body named the National Medical College (NMC) that later metamorphosed into the National Postgraduate Medical College of Nigeria (NPMCN).^2^ The decree establishing the NMC was promulgated into law in September 1979 by former military Head of State, General Olusegun Obasanjo.^2^ Simultaneously, the West African Health Society established in 1976 the West African College of Physicians (WACP) that was to superintend postgraduate medical training in West African sub-region.^7^ It consists of the following faculties: Internal Medicine, Laboratory Medicine, Community Health, Family Medicine, Paediatrics and Psychiatry.^7^

Both the NPMCN under the Faculty of Psychiatry (FMCPsych) and the WACP have been responsible for producing specialists in psychiatry since their inception in 1979 and 1976, respectively, leading to the award of Fellowship of NPMCN under the Faculty of Psychiatry (FMCPsych) and Fellowship of West African College of Physicians (FWACP) Psychiatry, respectively.

Under the control of the Nigerian Medical Council (Postgraduate Examinations), today’s Faculty of Psychiatry of NPMCN was founded under the administrative leadership of the first board that was chaired by late Prof. Tolani Asuni and Dr A. Anumonye as the secretary in 1974 well before the promulgation of the NPMCN into existence in 1979 by Decree number 67.Between the inception of the NPMCN and WACP and now, about 250 indigenous specialist psychiatrists have been produced till date and about 200 psychiatric trainees are presently undergoing specialist training as supervised by both colleges. Specialists are appointed into the position of Consultant Psychiatrist in Nigeria with either Fellowship of NPMCN under the Faculty of Psychiatry (FMCPsych) or with the Fellowship of West African College of Physicians under the Faculty of Psychiatry (FWACP) or their equivalent which could be the Member of the Royal College of Psychiatry (MRCPsych) with a number of years of practicing experience.

To obtain the fellowship of the two colleges, one would need to undergo three stages of the examinations – the primary examination that examined related basic medical sciences, Part 1 examination that tests the core clinical expertise of the specialty and Part 2 fellowship examination that is usually made up of oral examination and defence of a dissertation or thesis before award of the fellowship of either colleges. Both colleges collaborate in the designing of training and development of curriculum. The West African College serves the countries in the West African sub-region and the NPMCN is strictly for Nigeria. The fellowships of the two colleges are equivalent in Nigeria for the purpose of employment and practising as a specialist.

## Recent developments

The NPMCN in the Faculty of Psychiatry in recent years approved Post-Fellowship sub-specialty trainings in Child and Adolescent Psychiatry and Old Age Psychiatry in Nigeria.^8^

## Association of Psychiatrists in Nigeria

All the psychiatrists practising in Nigeria are bound together under the umbrella of the Association of Psychiatrists in Nigeria (APN) inaugurated in 1970.^9^ This association consists of full-fledged psychiatrist and associate members, who are usually psychiatric trainees and some allied professionals.^9^ The Association of Child and Adolescent Psychiatry and Allied Professionals in Nigeria (ACAPAN) was registered recently under the umbrella of APN.

## Current controversies regarding postgraduate medical training in psychiatry in Nigeria

Around 2008, the Nigerian University Commission (NUC), a body that regulates university education in Nigeria, issued a directive that possession of PhD degree would now be a prerequisite for promotion to a professorial level in a university setting as against just the fellowships of either colleges that are acceptable for postgraduate medical teachers in university settings in Nigeria. This directive was vehemently rejected by officials of both the NPMCN and the WACP and surgeons who argued that medical training, and more so postgraduate medical training, is a hands-on bedside training that must be combined with didactic lectures along with research competence unlike PhD that is mostly classroom taught courses and research. And there is no way a PhD degree can replace the standard offered by the level of training competence afforded by fellowships of either colleges for the purpose of postgraduate medical training in Nigeria^10^ Since then, this controversy has continued raging and lately making the NPMCN bow under pressure to include alongside her award of Fellowship a Doctor of Medicine (MD) degree that could be deemed equivalent to PhD in other academic settings. Prior to the introduction of MD degree as a postgraduate medical degree, the degree being awarded to individuals who completed undergraduate medical training in Nigeria is Bachelor of Medicine or Bachelor of Surgery (MBBS or MBChB) depending on the particular university.

The curriculum for the MD degree and training design which would be incorporated into the structure of normal Residency Training Programme leading to the award of Fellowship is presently going through finishing touches.^11^ The fact whether this step would improve the quality of postgraduate medical teachers in the university environment remains to be seen. It is the opinion of many specialist medical professionals that the change being accommodated by the NPMCN is just a cosmetic way of bowing to political pressure because Decree number 67 of 1979 has specified and defined the role of NPMCN as a body that would regulate the training and practice of postgraduate medical education in Nigeria. Whilst it is argued that undergraduate medical training may be under the supervision of or housed by a college in the university, postgraduate medical training, on the other hand, can actually be set up in any accredited health service institution across the country and not necessarily under the supervision of a university regulatory authority (NUC).

## Conclusions

This historical perspective is important at this present time in view of the current controversies arising in the university environment in Nigeria based on the new directive by the NUC, requiring every postgraduate medical teacher in a university environment to have a doctorate degree (PhD or MD) before being qualified for promotion to a professorial level in a Nigerian university setting. A moratorium was recently given by the NUC to postgraduate medical teachers in the university environment to obtain a PhD or MD degree by 2025 to be able to qualify for the promotion to the level of a university professor.

## References

[1] Bakare MO, Munir KM. Nigeria and autism. In: Volkmar F, editor. Encyclopedia of autism spectrum disorders. New York, NY: Springer; 2018. 10.1007/978-1-4614-6435-8

[2] National Postgraduate Medical College of Nigeria (NPMCN). Commemorative compendium for 50th anniversary of commencement of postgraduate medical training in Nigeria. Nigeria: National Postgraduate Medical College of Nigeria, Ijanikin, Lagos, Nigeria: 2019.

[3] Sidandi P, Gittelman M. Obituary: Prof. Thomas Adeoye Lambo, OBE (1923–2004) Nigerian Psychiatrist Former Vice Director General, World Health Organization. Int J Ment Health. 2004;33(4):81. 10.1080/00207411.2004.11043383

[4] Oyebode F. Obituary: Tolani Asuni, formerly Professor of Psychiatry, University of Ibadan, Nigeria. Psychiarist. 2011;35(12):478. 10.1192/pb.bp.111.037440

[5] Gureje O. Psychiatry in Nigeria. Int Psychiatry [serial online]. 2003 [cited 2020 Jul 31];1(2):10–12. Available from: https://europepmc.org/article/med/31507663 PMC6735237 31507663

[6] Medical and Dental Council of Nigeria (MDCN). About us [homepage on the Internet]. 2020 [cited 2020 Aug 19]. Available from: https://www.mdcn.gov.ng/page/about-us/mdcn-act-other-regulation

[7] West African College of Physicians (WACP). About us [homepage on the Internet]. 2020 [cited 2020 Jul 31]. Available from: https://wac-physicians.org/pages/about-us

[8] National Postgraduate Medical College of Nigeria (NPMCN). Faculty of Psychiatry [homepage on the Internet]. 2020 [cited 2020 Jul 31]. Available from: http://npmcn.edu.ng/faculties/faculty-of-psychiatry/

[9] Association of Psychiatrists in Nigeria (APN). About us [homepage on the Internet]. 2020 [cited 2020 Jul 31]. Available from: http://www.apn.org.ng/pgs/aboutus

[10] University World News. Nigeria – Top medical college rejects PhD directive [homepage on the Internet]. University World News; 2008. Available from: https://www.universityworldnews.com/post.php?story=20081010091735732

[11] National University Commission (NUC). NPMCN Collaborates with NUC in Medical Training; National University Commission. [homepage on the Internet]. 2020 [cited 2020 Jul 31]. Available from: https://www.nuc.edu.ng/npmcn-collaborates-with-nuc-in-medical-training/

